# Results from the Registry of Atrial Fibrillation (AFABE): Gap between Undiagnosed and Registered Atrial Fibrillation in Adults—Ineffectiveness of Oral Anticoagulation Treatment with VKA

**DOI:** 10.1155/2015/134756

**Published:** 2015-07-01

**Authors:** Anna Panisello-Tafalla, Josep Lluís Clua-Espuny, Vicente F. Gil-Guillen, Antonia González-Henares, María Lluisa Queralt-Tomas, Carlos López-Pablo, Jorgina Lucas-Noll, Iñigo Lechuga-Duran, Rosa Ripolles-Vicente, Jesús Carot-Domenech, Miquel Gallofré López

**Affiliations:** ^1^EAP Tortosa 1-Oest, Institut Català Salut, SAP Terres de l'Ebre, Health Department, Generalitat de Catalunya, CAP Roquetes, 43520 Tortosa, Spain; ^2^EAP Tortosa 1-Est, Institut Català Salut, SAP Terres de l'Ebre, Health Department, Generalitat de Catalunya, CAP Temple, 43500 Tortosa, Spain; ^3^Miguel Hernández University, 03202 Elche, Spain; ^4^EAP-Camarles-Aldea-Ampolla, Institut Català Salut, SAP Terres de l'Ebre, Health Department, Generalitat de Catalunya, CAP Ampolla, 43895 Tortosa, Spain; ^5^EAP Tortosa-2-Oest, Institut Català Salut, SAP Terres de l'Ebre, Health Department, Generalitat de Catalunya, CAP Xerta, 43592 Tortosa, Spain; ^6^Molecular Biology and Research Section, Hospital de Tortosa Verge de la Cinta, IISPV, IDIAP, 43500 Tortosa, Spain; ^7^EAP Deltebre, Institut Català Salut, SAP Terres de l'Ebre, Health Department, Generalitat de Catalunya, CAP Deltebre, 43580 Tortosa, Spain; ^8^Department of Cardiology, Hospital de Tortosa Verge de la Cinta, IISPV, 43500 Tortosa, Spain; ^9^Department of Systems Management, Hospital de Tortosa Verge de la Cinta, Institut Català de la Salut, 43500 Tortosa, Spain; ^10^Pla Director de la Malaltia Vascular Cerebral de Catalunya, Department Salut de la Generalitat de Catalunya, 08005 Barcelona, Spain

## Abstract

*Objective*. This study aimed to examine the effectiveness of the use of oral anticoagulation (OAC) medication, recommended by national guidelines for stroke prevention but reportedly underused in AF patients with moderate to high stroke risk. *Method*. A multicentre and cross-sectional study of undiagnosed AF among out-of-hospital patients over 60 years old was carried out, visiting 3,638 patients at primary health centres or at home for AF diagnosis using the IDC-10 classification. The main outcome measures were CHA_2_DS_2_VAS_C_, HAS-BLED scores, cardiovascular comorbidity, pharmacological information, TTR, and SAMe-TT2R2 scores. *Results*. The main findings were undiagnosed AF in 26.44% of cases; 31.04% registered with AF but not using OAC despite 95.6% having a CHA_2_DS_2_VAS_C_ ≥ 2 score; a risk of bleeding in important subgroups using OAC without indication (37.50% CHA_2_DS_2_VAS_C_ < 2 score); the use of OAC with TTR < 60% (33.1%), of whom 47.6% had a HAS-BLED score ≥3. Thus, 35.4% of the expected AF prevalence achieved an optimal time in the therapeutic range. *Conclusions*. The expected AF prevalence was 10.9% (*n* 5267), but the registered prevalence was 7.5% (*n* 3638). Only 35.04% (CI = 95%, 33.7–36.3) of AF patients treated with vitamin K antagonists (VKAs) achieve the goal of TTR > 60%.

## 1. Introduction

Atrial fibrillation (AF) is a common cardiac arrhythmia [1] that affects 1-2% of the general population and accounts for one-third of hospitalizations for heart rhythm disturbances. The risk increases with age [2]. With the aging of the population, the number of patients with AF is expected to increase 150% in the next four decades, with more than 50% of patients being over the age of 80. This increasing burden from AF will lead to a higher incidence of stroke, as patients with AF have a five- to sevenfold greater risk of stroke than the general population [3, 4]. Strokes secondary to AF have a worse prognosis than in patients without arrhythmia. In addition, the costs of managing AF patients and its complications have been well documented and are high [5]. This will have serious implications for the planning of health and welfare systems, not only because of predictions of a continuous increase in AF prevalence [3, 6, 7] given the close association between arrhythmia and aging, but also because of the current cost constraints due to the economic context.

Due to the associated increased morbidity, mortality, and cost, challenges in the identification of patients at risk for thromboembolic events from AF must be addressed. AF is often only detected with the onset of severe AF-related complications such as stroke or heart failure [8, 9]. Although national guidelines recommend the use of oral anticoagulation (OAC) medication for stroke prevention and there is clear evidence of the effectiveness of vitamin K antagonist (VKA) therapy in patients with AF [10], the literature consistently reports its underuse in AF patients with moderate to high stroke risk [1, 11]. This underutilization imposes a substantial clinical and economic burden on healthcare systems. Finally, the percent time in therapeutic INR range (TTR) has been used to evaluate the effectiveness of VKA therapy as a quality measure, but there is a general lack of quality measurement in OAC use. Data show that if the TTR is < 50%, the result is actually worse than not using any warfarin at all, whereas when the therapeutic range is at least 70%, the likelihood of stroke or systemic embolism is very small [12]. This paper highlights the results of clinical practice in patients with AF, focusing on the assessment of results in the rates of appropriate use of and patient adherence to OAC treatment plans administering VKAs (warfarin/acenocoumarol) beyond simply examining the percentage of AF patients treated with OAC.

The aim of the study is to document the quality of anticoagulant control in primary care, considering the potential impact of undiagnosed AF, the underutilization of VKAs, and results related to TTR. The challenges include compliance with performance measures, adherence to guidelines, adequate prevention, and early control of comorbidities that affect the progression of AF and associated risks, early initiation of treatment, and successful evaluation of the associated risks of bleeding, primary or recurrent stroke, and patient awareness and compliance [1, 8, 13].

## 2. Materials and Methods

The AFABE [8, 13] study is a cross-sectional, multicentre study of undiagnosed AF among out-of-hospital patients over 60 years old attending primary care teams in the Terres de l'Ebre health area in Catalonia, north-eastern Spain, on 31 July 2014. The patients in the sample were registered with health centres and were visited there or at home for AF diagnosis according to the routine ICD-10 classification used in the primary care dataset for a revision of the electronic medical history. The variables for which data were collected are as follows.

*(1) Patient Identification Code*. It includes individualized TIS number (individual health card used in Catalonia).

*(2) Sociodemographic Information*. It includes age, gender, and place of residence.

*(3) Cardiovascular Information*. We described clinical comorbidities included in the cardioembolic CHA_2_DS_2_VAS_C_ rule [14, 15] (congestive heart failure; hypertension; age ≥ 75 years [doubled]; type 2 diabetes; previous stroke, transient ischemic attack, or thromboembolism [doubled]; vascular disease; age 65–75 years; sex category) and HAS-BLED (hypertension, abnormal renal/liver function, stroke, bleeding history or predisposition, labile INR, elderly, and drugs/alcohol concomitantly) [16, 17] codistribution representing bleeding risk among AF patients treated with VKAs. We considered them “previous” when they had been diagnosed and registered at least one month before the AF diagnosis and “later” when they had been diagnosed and registered simultaneously to or after AF diagnosis. Patients with a CHA_2_DS_2_VAS_C_ score ≥ 2 were categorized as high stroke risk and those with a HAS-BLED score ≥ 3 were categorized as high bleeding risk. We studied the age-specific incidence of all AF-related annual stroke rates, extrapolating average CHA_2_DS_2_VAS_C_ score values to the population and projecting future numbers [18, 19].

*(4) Pharmacological Information*. It includes drugs assigned as clinical treatment for all conditions including AF; antiarrhythmic agents received as a rhythm control strategy (class I/class III), with or without rate control strategy (class II/class IV, digital) and/or antithrombotic treatment; OAC treatment with VKAs (warfarin/acenocoumarol) or NOAC therapy and/or antiplatelet treatment and/or angiotensin-converting enzyme (ACE) inhibitors and/or statins. We used the concept “polymedication” (the prescription of at least 10 different medications simultaneously) to seek a possible relation with the percent time in therapeutic INR range.

**Figure 1:**
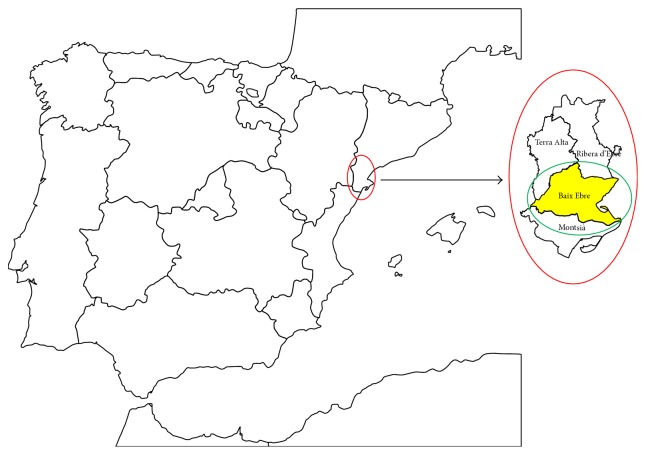
Current territory study Map. “Ebro Lands” is located in the southwest of Catalonia, in the southern part of river Ebre, and formed by four regions: Baix Ebre, Montsià, Terra Alta, and Ribera d'Ebre (all in red circle). AFABE study (Baix Ebre, in green circle). The figure shows the relationship between the subjects in the previous study AFABE and those ones included in the current study.

*(5) Diagnosis Dates*. AF incidence, cardiovascular comorbidities, and death dates (all-cause mortality) are registered in patients' electronic medical PC. All the diagnostics were defined in the patient dataset using the ICD-10 classification. As this was a retrospective study of confirmed AF, we did not include cases with a changed diagnosis of AF or with unconfirmed AF. The* registered AF prevalence* included people who were* a case with a diagnosed and registered AF in their public health primary care electronic medical history* according to the ICD-10 routine classification used in the primary care dataset for a revision of the electronic medical history. Based on the census of 2011, the* expected AF prevalence* was calculated using the data obtained in the AFABE study [8, 13]. The AFABE (Baix Ebre) study was a sample of the current population (Figure 1).

*(6) INR Control*. The TTR for individual patients was estimated by Rosendaal method [20], using linear interpolation to assign an INR value to each day between two successively observed INRs. If the sampling interval exceeded 60 days, values were not interpolated. Patients with less than three consecutive INRs were excluded to achieve a meaningful estimation of the TTR. Likewise, the first two weeks of INRs were excluded from the analysis for patients who began warfarin treatment. Adult patients with AF who used warfarin for a 12-month period with no gap of > 60 days between visits were identified and the records collected were analysed. We considered the* average time* in therapeutic range to be lower if it was < 60%. VKAs (warfarin/acenocoumarol) are the anticoagulant therapy of choice in Catalonia for patients with AF who are at risk of stroke. The patients were stratified according to their proportion of time in range.

*(7) SAME-TT*_*2*_ *R*_*2*_ *Score [21]*. It includes sex, age (<60 years), medical history (at least two of the following: hypertension, diabetes, coronary artery disease/myocardial infarction, peripheral arterial disease, congestive heart failure, previous stroke, pulmonary disease, and hepatic or renal disease), and treatment (interacting drugs, e.g., amiodarone for heart rhythm control) [all 1 point], as well as current tobacco use (2 points) and race (non-Caucasian; 2 points). The SAME-TT_2_R_2_ score was calculated for all patients with a diagnosis of AF, but as it makes a simple prediction of which AF patients are likely to do well on VKAs (with an average time in therapeutic range ≥ 60%), the TTR percentage included just those patients using VKAs. We tested the hypothesis that the new SAME-TT_2_R_2_ score was a predictor for good average time in therapeutic range and, second, that this would translate into adverse events in a “real-world” cohort of patients with AF.

### 2.1. Statistical Analysis

In the descriptive analysis, the data for categorical variables are expressed as number of cases and percentages and the data for continuous variables are expressed as means with standard deviations and/or IC95%. Categorical variables were compared using a *χ* ^2^ test or Fisher's exact test depending on the application conditions. Continuous variables were compared using Student's *t*-test or the Mann-Whitney *U* test depending on the normal distribution assumption. Normal distribution was checked using the Shapiro-Wilk test. A logistic regression analysis was performed to find possible risk factors that characterize the population with AF. A *P* value of less than 0.05 was considered to indicate statistical significance in all statistical tests. The analysis was carried out using the SPSS statistical software package (version 19).

## 3. Results

**Figure 2:**
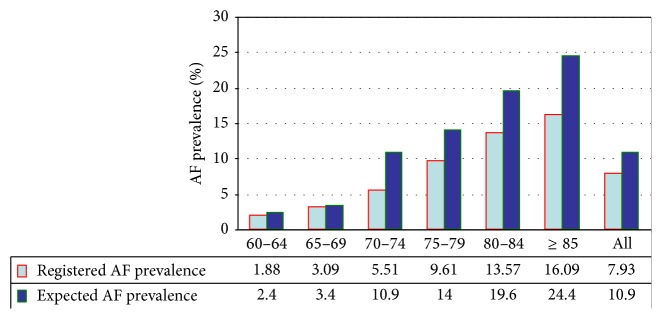
Registered prevalence distribution versus expected prevalence of AF by age groups. The expected AF prevalence [13] was 10.9% (*n* 5267), but the registered prevalence was just 7.5% (*n* 3638). The rate of undiagnosed AF for subjects over 60 years old was 3.4%, representing 31% of the overall AF prevalence in our study, compared to a percentage between 25% and 35% in other studies [22–26].

The Ebro Lands study population (Figure 1) comprised 48,325 ≥ 60 years old in the census of the territory. Of these, 92% use primary care services. Their mean age was 78.7 years (SD = 7.3) and 53.6% were men. We examined 3,638 (1,689 female, 1,949 male) AF patients registered for AF diagnosis. The registered AF prevalence was 7.5% (CI = 95% 7.3–7.7); when stratified by gender and age (Figure 2), the groups progressively increased. The average age at AF diagnosis was 73.65 ± 8.0 years; 75% were ≥ 75 years.

Table 1 shows the group baseline data and cardiovascular risk factors in AF patients and the average CHA_2_DS_2_VAS_C_ and HAS-BLED scores; these are stratified by age in Table 2. A high prevalence of cardiovascular risk factors (CVRF) was found for hypertension (HTA, 77.1%) and diabetes mellitus type 2 (DM2, 26.5%). Men had significantly more prevalence of DM2, previous stroke, vascular diseases, and smoking. The average CHA_2_DS_2_VAS_C_ score was 3.6 and 95.6% of subjects had a CHA_2_DS_2_VAS_C_ score ≥ 2. The older the patient (increasing up to 85 years), the higher the CHA_2_DS_2_VAS_C_ score.

The average HAS-BLED score was registered in 69.5% of patients. The average score was 2-3 and 47.6% of subjects treated with VKAs had HAS-BLED ≥ 3. The proportions of subjects with abnormal renal function and abnormal hepatic function were 14.4% and 15.1%, respectively. Risk factors related to a history of or predisposition to bleeding (2.3%) and chronic concomitant use of antiplatelet and/or anti-inflammatory (5.2%) treatment were less frequent. The older the patient (increasing up to 85 years), the higher the HAS-BLED score.

Before the AF diagnosis, 36.8% (CI = 95%, 30.8–42.7) had been diagnosed with some cardiovascular complication (CVC). Almost half of the overall CVCs were ischemic cardiomyopathy (24.2%) and ischemic stroke (23.2%). The incidence of ischemic cardiomyopathy was significantly higher among men (*P* = 0.031), while the incidence of ischemic stroke was similar among men and women (*P* = 0.612). There were no differences in the overall incidence of CVC by gender. Patients who had suffered ischemic stroke or ischemic cardiomyopathy previously were at greater risk (OR = 2.63) of suffering AF than those who had not. Mortality was higher (*P* = 0.05) among those ones who had been diagnosed with ≥ 2 CVCs before the AF. On other hand, mortality was significantly lower among those who were treated with statins (*P* = 0.032).

**Figure 3:**
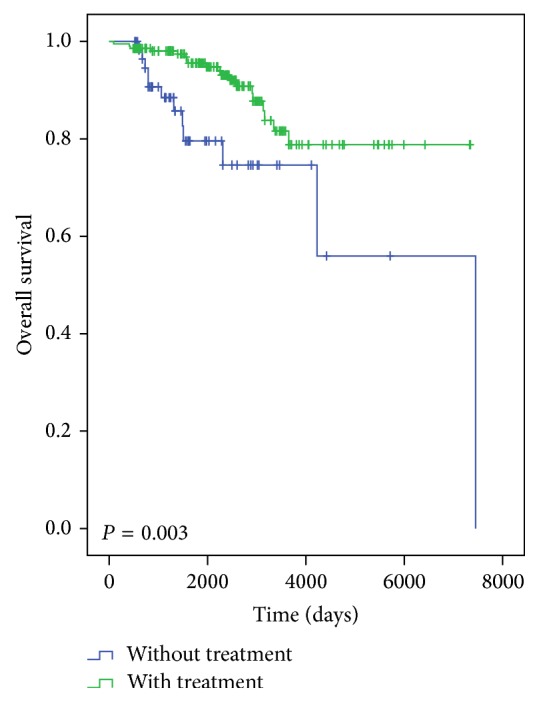
Survival curve and treatment with oral anticoagulant agents. The main predictor of mortality was nontreatment with OAC, with significantly lower mortality in patients treated with VKA instead of antiplatelet (*P* = 0.003). This figure has been published previously [27]. The overall percentage of patients not treated with OAC was 26.9% (CI = 95%, 22.7–30.9). Mortality was higher (*P* = 0.05) among those ones who had been diagnosed with ≥ 2 CVCs before the AF. On other hand, mortality was significantly lower among those who were treated with statins (*P* = 0.032).

Simultaneous to or after AF diagnosis, 28.6% (CI = 95%, 38.1–50.3) were diagnosed with new vascular complications. The most frequent vascular complication was congestive heart failure (CHF, 46.7%), the incidence of which was significantly higher among women (*P* = 0.037). The five-year survival rate with a diagnosis of CHF is lower (0.69 ± SD 0.09) than when there is no CHF present (0.96 ± DE 0.01). The main predictor of mortality is a nontreatment with OAC, with significantly lower mortality in patients treated with OAC (*P* = 0.003) versus antiplatelet treatment (Figure 3). There were no differences in the overall incidence of cardiovascular complications by gender.

The prevalence of ischemic stroke and AF was 15.07%. There were 438 incidents of AF per year, 9.1 (CI = 95%, 8.2–10.0)/1000 patients ≥ 60 years old per year. Of 565 incidents of ischemic stroke, 359 (63.5%) occurred at ≥ 80 years (Table 2). Overall, the incidence of AF-related strokes was 1.11 (CI = 95%, 0.9–1.5)/1000 AF patients ≥ 60 years old per year. The numbers of AF-related strokes at age ≥ 80 years were double the average incidence (Table 1). In the Cox regression, after adjusting for age, gender, number of cardiovascular complications before and after AF diagnosis, OAC treatment, antiplatelet treatment, and other specific treatments, the only variable with a protective value against mortality was antithrombotic treatment (HR = 0.344, CI = 95%, 0.163–0.728) (Figure 3).

**Figure 4:**
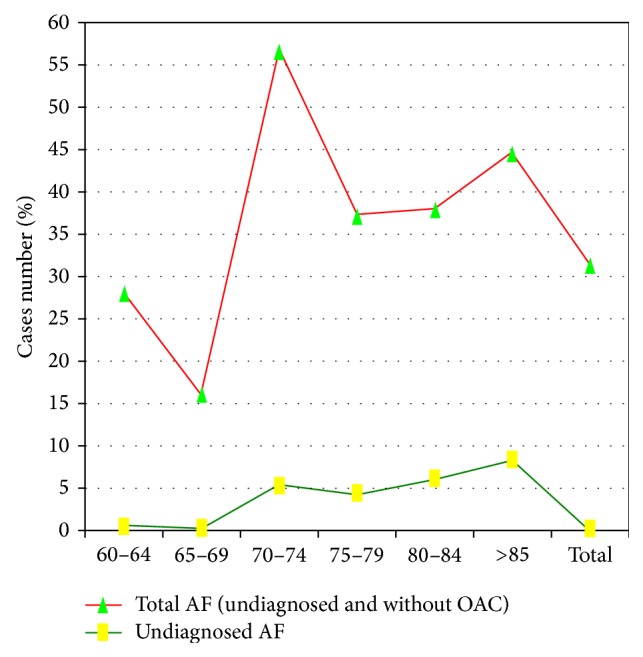
Total and undiagnosed atrial fibrillation not treated with OAC. In terms of the expected AF prevalence [9, 10], the percentage of undiagnosed AF rises with age. It is notable that the greatest proportion of AF cases was in patients aged 70–74 years (51.2%; CI = 95%, 47.8–54.5) with unknown AF and no OAC treatment.

The overall percentage of patients not treated with OAC was 26.9% (CI = 95%, 22.7–30.9). In all, 4.2% were treated with NOACs (apixaban, dabigatran, or rivaroxaban) and 18.9% with antiplatelet drugs. In terms of the expected AF prevalence [9, 10], the percentage of undiagnosed AF rises with age. Approximately 1,630 AF patients could have remained undiagnosed and the overall average percentage without OAC treatment was 31.0% (CI = 95%, 29.7–32.3). It is notable that the greatest proportion of AF cases was in patients aged 70–74 years (51.2%; CI = 95%, 47.8–54.5) with unknown AF and no OAC treatment. The percentage of AF with no OAC treatment rises with age (Figure 4).

The percentage of patients with time in therapeutic range (TTR) < 60% was 33.1% (CI = 95%, 30.5–35.6) for those using VKAs. This research identified a high rate of patients with anticoagulant therapy in primary healthcare (>90%). Most patients take coumarins and the quality of OAC control is reasonably high. In all, 50.5% had a SAME-TT_2_R_2_ score ≥ 2 and the percentage gradually increased in patients from 60 to 64 years (36.8%) up to > 85 years (57.6%). Of these, 54.6% had TTR < 60% and the ROC curve results were 0.48 (CI = 95%, 0.46–0.50), sensitivity 0.62, and specificity 0.29.

**Figure 5:**
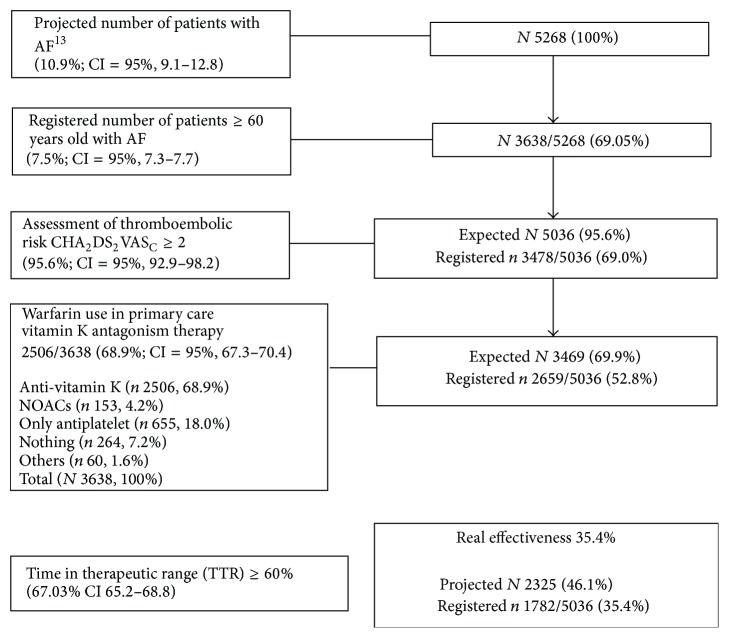
Draft* study outline*: effectiveness of anticoagulant control. The percentage of undiagnosed AF should be added to the percentage of known AF not treated with OAC, representing 40–50% [28] of the overall AF. Clearly, if we add the TTR results, this percentage lowers even more. According to the panel data [1], only 35.4% (CI = 95%, 33.7–37.3) of AF patients treated with VKAs achieved the goal of optimal effectiveness in order to secure clinical benefits.

It is possible to improve patients' control of their VKA serum levels in various ways, but clearly it is necessary to address risk conditions reflecting poor anticoagulation control and labile INRs among patients with AF given that OAC treatment seems to depend solely on these. This study has found a gap (30.9%) between expected and registered AF prevalence and registered AF with no use of OAC treatment (26.9%) despite 95.6% having a score of CHA_2_DS_2_VAS_C_ ≥ 2 and there being a group at special risk (70–74 years) of whom 47.6% had a score ≥ 3 for HAS-BLED. Furthermore, there is a risk of bleeding in important subgroups using OAC without indication (37.50%, CHA_2_DS_2_VAS_C_ < 2). In this study, one-third (33.1%) of AF patients using OAC showed a time in therapeutic INR range < 60%; the older the patient, the higher the risk of TTR < 60%. According to the panel data [1], only 35.4% (CI = 95%, 33.7–37.3) of AF patients treated with VKAs achieved the goal of optimal effectiveness in order to secure clinical benefits (Figure 5).

## 4. Discussion

This paper focuses in particular on the overall prevalence of AF increasing with age and its related possible consequences, that is, more undiagnosed AF. Furthermore, the potential increase in the percentage of AF not treated with OAC carries the foreseeable threat of an increase in cardiovascular morbidity and associated costs, primarily caused by ischemic stroke, stroke, and CHF with the requirement for chronic use of medication. Our main conclusion, unlike other studies, is not much the relative underuse of the VKA treatment in high risk AF patients but the low efficiency resulting from the association between its underuse and undiagnosed AF.

The rate of undiagnosed AF for subjects over 60 years old was 3.4%, representing 31% of the overall AF prevalence in our study, compared to a percentage between 25% and 35% in other studies [22–26]. Although it is practically impossible to reduce the prevalence of undiagnosed AF to zero, it is possible try to reduce it. New external devices that register prolonged intermittent arrhythmia have substantially improved the detection of silent paroxysmal AF in patients with recent ischemic stroke/transient ischemic attack [29]. Nevertheless, until these new external devices can be used more widely, ECG combined with reviews of medical history will continue to be the most feasible noninvasive strategy for identifying individuals with AF in epidemiological studies. The key issue, however, is not which test is best for diagnosing AF or how to undertake an effective screening procedure, but it is rather the appropriate measurement of results and achieving optimal effectiveness.

The prevalence of AF is high and rising. This work has paid particular attention to the alarming increase in overall AF prevalence, especially in people >70 years, the prevalence of AF being > 20% at age ≥ 80 years. Similar results have been found [30] suggesting that the total number of people with AF in a practice could be around 10% of the number of people aged 60 and over. From the sixties to over the eighties the prevalence increases by 10 times [13]. The population over 80 in the last 30 years has grown by approximately 66%, representing an increase from 3.5% to 5.9% of the total population. At the current incidence rates, the numbers of AF-related embolic events at age ≥ 80 years will treble by 2050, with most events occurring in this age group. Approximately 15% to 25% of ischemic strokes are attributed to AF [3, 4], a similar proportion to that in our results. Among patients ≥ 80 years with AF, the effectiveness of anticoagulation treatments should be a major public health priority [31], although the impact of population aging on rates of AF-related ischemic events is uncertain.

The percentage of undiagnosed AF should be added to the percentage of known AF not treated with OAC, representing 40–50% [28] of the overall AF. Clearly, if we add the TTR results, this percentage lowers even more. Despite the effectiveness of OAC treatment, recent literature reviews and studies have pointed to the fact that the current practice does not follow published guidelines with undertreatment in spite of the current evidence of the benefits of anticoagulation therapy for AF in patients with moderate to high CHA_2_DS_2_VAS_C_ risk scores [32], resulting in a substantial occurrence of preventable ischemic stroke [33]. The panel data also show that geriatric patients should receive OAC treatment as a rule, unless a comprehensive neurological and geriatric assessment provides sound reasons for refraining from such treatment [1]. Patients with a CHA_2_DS_2_VAS_C_ score ≥ 2 should receive anticoagulation even if at high risk of falls. The risk scores, CHA_2_DS_2_VAS_C_ and HAS-BLED, rise with age up to 85 years, but, as the risk of stroke increases, the rate of anticoagulation use does not differ or decrease. This may be due to the concerns of providers regarding the risk of bleeding and the risk-benefit trade-off of treatment for higher-risk populations.

The reasons for the underuse of anticoagulation are poorly understood. There is a complex interaction between patient-, physician-, and healthcare system-related factors [34], in which the need to maintain the INR level within the therapeutic range and the difficulty of doing so probably play a major role. These findings suggest that providers are using factors other than clinical risk stratification tools to guide anticoagulation decisions in high risk patients. The optimal approach to stroke prevention in geriatric patients with AF has not adequately been clarified. Despite their high risk of stroke and the clear indication for anticoagulation treatment according to established risk scores, in practice, this treatment is often withheld from geriatric AF patients because of comorbidities and comedications, concerns regarding low treatment adherence, or fear of bleeding events, in particular due to falls. The factors associated with reluctance in prescribing anticoagulants are increasing age, female sex, treatment at a nonneurological department, worsening disability, dementia, high risk of bleeding, terminal disease, or patient's choice [27, 35]. We need more research on tools such as the SAME-TT_2_R_2_ score, including investigations in different local cultural conditions, leading to new quality criteria based on the results.

It is important to understand whether there are appropriate reasons for the apparent underuse of warfarin therapy in the elderly [9, 13, 36, 37] that may include the risk of bleeding and falls, nonproper study of population subjects, concerns over bleeding risk, aggressiveness in achieving the INR point target, comorbidities, exclusion or limitations based on a protocol for prescribing NOACs, or polypharmacy. The data suggest that cardiologists and primary care physicians have different conceptualizations of stroke and bleeding risks and primary care physicians may be less likely to prescribe OACs.

A prior history of falls has been associated with increased risk of stroke/thromboembolism, bleeding, and mortality, but not haemorrhagic stroke in the presence of anticoagulation [38]. It is necessary to determine potential outcomes of haemorrhagic stroke in terms of mortality and loss of autonomy and consider these risks as an essential element in the planning of home care, including the prevention of accidents. However, fall risk in elderly patients on antithrombotic therapy was studied in a meta-analysis which demonstrated that elderly patients taking warfarin would have to fall approximately 300 times per year for the risk of bleeding complications from falling to outweigh the benefits of embolic stroke prevention [39]. A moderate risk of falling in the elderly population should not be an absolute contraindication for anticoagulation treatment [40, 41]. All patients with a history of falls should be evaluated thoroughly to determine the causes.

Epidemiologically and clinically, an increase in the percentage of haemorrhagic stroke can be observed, progressing from 7.9% (2006–2008) to 14.8% [42] (December 2013), a statistically significant difference (*P* < 0.001). There has been an increase in patients on warfarin and 40% of haemorrhagic strokes occurred at ≥ 80 years. In our study, 47.6% of AF patients treated with VKAs scored ≥ 3 for HAS-BLED. A tendency towards an increasing frequency of stroke has been observed for increasing bleeding risk within cardioembolic risk categories and vice versa [43]. In addition, polypharmacy is an important marker of both multimorbidity and burden of treatment. Of the people with a stroke, the proportion that had one or more additional morbidities present was almost twice that in the control group [44]. We propose its inclusion as variable for analysis in the SAME-TT_2_R_2_ score.

Ultimately, the quality of OAC treatment with warfarin is measured primarily by TTR. According to guidelines [1], if a TTR of more than 70% cannot be maintained, treatment with NOACs should be considered. Internationally, studies of the quality of OAC treatment in general practice have consistently shown poor results [45], but we found a mean c-TTR of 67.03%, similar to others [36, 46] in general practice, which suggests that GPs provide OAC treatment of good quality. However, the TTR calculations do not include electronic data capture of INRs to assist GPs in monitoring TTR and undertaking appropriate follow-up measures.

Looking at the NOACs together, there is evidence of a significant reduction in intracranial haemorrhage [47] and also in stroke or systemic embolism. These are safer and less expensive socially [48] and facilitate management in the geriatric population with AF: no INR monitoring is needed, there is easier bridging during interventions, and there are fewer risks and better results. Furthermore, based on the data available, they exhibit a better benefit-risk ratio compared to VKAs. Drugs with predominantly nonrenal elimination are safer in geriatric patients and should be preferred [39]. We should consider NOACs an interesting option in slowing down the current evolution of approximately 30% fewer strokes every year. This management decision is often complex and involves taking into account contraindications, financial constraints, patient preferences, and cost-benefit analyses. NOACs are more likely to be cost-effective options in settings with poor warfarin management than in settings with better anticoagulation control, where they may not represent good value for money [49].

Our regional findings reflect the care provided by a limited set of investigators in any geographic region. These differences may explain, in part, the current divergence of anticoagulation treatment decisions from guideline recommendations, but we believe that it is essential to achieve quality assurance information on anticoagulation therapy at a local level beyond just the treatment coverage. In particular, we consider it very important to determine the expected number of AF patients according to the demographic characteristics of the beneficiary population as undervaluation results from solely describing AF patient numbers with OAC treatment. Also, access to electronic support tools in clinics using TTR for monitoring could lead to an increase in quality and would allow for ascertaining in detail how the GPs managed all the aspects of the treatment [50]. The recent approvals of several new, novel OAC agents with a benefit/risk profile that represents an important advance over VKA prophylaxis [51] have given rise to great expectations in the management of these patients but also new doubts. The main limitations to their general use are the lack of data for some subgroups of frail patients and the lack of availability of specific antidotes and especially their high cost.

## 5. Study Limitations

As this is a study of subjects requesting primary care attention (at health centres, their own homes, or their care or nursing homes), it is possible that a higher frequency of AF patients or patients with AF risk factors could have produced an artificial increase in the prevalence described. The strengths of the present study include a population-based design and its reflection of routine clinical practice. Minimal exclusions were employed as the exclusion of patients with missing data would potentially have introduced selection bias. The risk of referral bias was low as it can be assumed that all patients with acute symptoms of stroke are referred to the public healthcare system if hospitalized. The weaknesses of the present study include the retrospective design and the risk of misclassifications during data collection in routine clinical settings.

## 6. Conclusions

The expected AF prevalence was 10.9% (*n* 5267), but the registered prevalence was just 7.5% (*n* 3638). Although the “gold standard” for anticoagulation is warfarin, only 68.9% (*n* 2506) of patients were treated with VKAs and only 67.3% attained a TTR > 60%. A relatively high rate of patients with anticoagulant therapy in primary healthcare has been found in this research, but the INR control remains suboptimal. Thus, only 35.4% of the expected AF prevalence achieved an optimal TTR. It seems clear that the providers of care and the systems within which they work have a profound effect on the quantity and quality of anticoagulation treatment.

## Figures and Tables

**Table 1:** Subjects' baseline data and cardiovascular risk factors in AF patients and the average CHA_2_DS_2_VAS_C_ and HAS-BLED scores.

Subjects	
Registered AF prevalence	(*N* 3638)
	(7.5%; CI = 95%, 7.29–7.76)
Women (%)	1688 (46.4)
Mean age (years)	78.7 ± 7.30
Age ≥75 y (%)	74.5
Hypertension (% CI = 95%)	77.1 (CI = 95%, 71.9–82.3)
Diabetes mellitus (% CI = 95%)	26.5 (CI = 95%, 21.1–32.01)
Vascular disease (% CI = 95%)	14.7 (CI = 95%, 10.35–19.16)
Previous stroke/TIA (% CI = 95%)	17.4 (CI = 95%, 12.65–22.03)
Heart failure (% CI = 95%)	22.8 (CI = 95%, 17.7–20.06)
Thromboembolism (% CI = 95%)	2.2 (CI = 95%, 0.27–4.15)
CHA_2_DS_2_VAS_C_ score ≥2 (% CI = 95%)	95.6 (CI = 95%, 92.9–98.2)
HAS-BLED score ≥3 (% CI = 95%)	47.6 (CI = 95%, 45.7–49.4)
TTR ≥ 60 (%)	67.0 (CI = 95%, 65.2–68.8)
Vitamin K antagonism (VKA) therapy (% CI = 95%)	68.9% (CI = 95%, 67.3–70.4)

**Table 2:** AF prevalence, TTR, and average CHA_2_DS_2_VAS_C_ and HAS-BLED scores stratified by age.

	60–64	65–69	70–74	75–79	80–84	>85	Total/mean
All (*N*)	9840	9690	7993	7146	6864	6792	48325
Men	4918	4756	3868	3331	2870	2543	22286 (46.1%)
Women	4922	4934	4125	3815	3994	4249	26039 (53.8%)

Registered cases							
Prevalence of AF							
*N*	185	300	441	687	932	1093	3638
(%)	(1.88)	(3.09)	(5.51)	(9.61)	(13.57)	(16.09)	(7.5%; CI = 95%, 7.29–7.76)
Men							1950 (53.6%)
Women							1688 (46.4%)

Expected cases (AFABE)							
Prevalence of AF							
*N*	236	329	871	1000	1345	1657	5268
(%)	(2.4)	(3.4)	(10.9)	(14.0)	(19.6)	(24.4)	(10.9%; CI = 95%, 9.1–12.8)

Absolute difference	−51	−29	−430	−313	−413	−564	−1630 (30.9%; CI = 95%, 28.6–32.2)

Average CHA_2_DS_2_VAS_C_ score	3.3%	8.9%	12.6%	24.5%	29.4%	21.2%	
(AFABE)	1.22	2.20	2.76	3.92	4.06	4.07	3.60 (CI = 95%, 3.41–3.79)

HAS-BLED ≥ 3 (%)	1.12%	14.6%	13.48%	24.71%	29.21%	16.85%	47.6% (CI = 95%, 45.7–49.48)

Total AF and no OAC (%)	27.5% (21.6–33.4)	15.8% (11.7–19.9)	51.2% (47.8–54.5)	32.8% (29.8–35.9)	31.9% (29.4–34.5)	36.1% (33.8–38.4)	31.04% (CI = 95%, 29.7–32.3)

TTR ≥ 60%	66.2	65.3	69.1	66.3	68.1	66.7	67.03% (CI = 95%, 65.2–68.8)

SAME-TT_2_R_2_ ≥2							
*N*	63	111	181	321	451	591	1805
(%)	(36.8%)	(40.0%)	(44.2%)	(49.1%)	(50.4%)	(57.6%)	50.5% (CI = 95%, 48.9–52.2)

Registered prevalence of stroke and AF							
*N*	18	39	46	103	140	219	565
(%)	(9.7%)	(13%)	(10.4%)	(14.9%)	(15.0%)	(20.0%)	15.53% (CI = 95%, 14.3–16.7)

Registered AF incidence/1000/year							
*N*	28	40	67	83	90	130	
*n*/1000/year	2.8	4.1	8.4	11.6	13.1	19.1	438
CI = 95%	(1.9–4.1)	(2.9–5.6)	(6.5–0.6)	(9.3–14.4)	(10.5–16.1)	(16.0–22.7)	9.1 (CI = 95%, 8.2–10.0)
